# Compensation claims after knee arthroplasty surgery in Norway 2008–2018

**DOI:** 10.1080/17453674.2020.1871187

**Published:** 2021-01-13

**Authors:** Per-Henrik Randsborg, Tommy Frøseth Aae, Ida Rashida Khan Bukholm, Anne Marie Fenstad, Ove Furnes, Rune Bruhn Jakobsen

**Affiliations:** a Department of Orthopaedic Surgery, Akershus University Hospital, Lørenskog, Norway;; b Department of Orthopedic Surgery, Health Møre and Romsdal HF, Kristiansund Hospital, Kristiansund, Norway;; c Norwegian System of Patient Injury Compensation, Oslo, Norway;; d Norwegian Arthroplasty Register, Department of Orthopaedic Surgery, Haukeland University Hospital, Bergen, Norway;; e Department of Health Management and Health Economics, Medical Faculty, University of Oslo, Oslo, Norway;; f Sports Medicine Institute, Hospital for Special Surgery, New York, USA;; g Department of Clinical Medicine, Faculty of Medicine, University of Bergen, Bergen, Norway

## Abstract

Background and purpose — Orthopedic surgery is one of the specialties with most compensation claims. We assessed the claims following knee arthroplasty surgery reported to the Norwegian System of Patient Injury Compensation (NPE) in light of institutional procedure volume.

Patients and methods — We collected data from NPE and the Norwegian Arthroplasty Register (NAR) for the study period (2008–2018). Age, sex, type of claim, and reason for compensation were collected from NPE, while the number of arthroplasty surgeries was collected from NAR. The treating hospitals were grouped by quartiles according to annual procedure volume. The effect of hospital volume on the likelihood of an accepted claim was estimated.

Results — NAR received 64,241 reports of arthroplasty procedures, of which 572 (0.9%) patients filed a claim for treatment injury. 55% of the claims were accepted, representing 0.5% of all knee arthroplasties. The most common reason for accepted claim was a hospital-acquired infection, in 28% of the patients, followed by misplaced implant (26%) and aseptic loosening (13%). The hospitals with the lowest annual volume (57 or fewer arthroplasties per year, first quarter) had a statistically significantly larger fraction of granted claims per procedures compared with other institutions.

Interpretation — The overall risk of ending up with compensation due to treatment error following knee arthroplasty was 0.5%. The risk of accepted claim was greater for patients operated in the lowest volume hospitals.

The number of knee arthroplasty procedures in Norway has increased over the last decade and is now over 7,000 per year (Ackerman et al. 2017, NAR 2020). About 1 in 5 patients receiving a TKA remains dissatisfied with the result (Gunaratne et al. 2017). Although serious complications are rare, infections, implant loosening, misplaced implants, residual pain, and other complications do occur, with potential detrimental results. To monitor the safety of implants and define the epidemiology of the procedures, the Norwegian Arthroplasty Register (NAR) was established in 1987 (Havelin et al. 2000). NAR provides a comprehensive overview of knee arthroplasties taking place in Norway. Compliance with the registry is 97.6% for primary TKA and 93.2% for revisions (Wiik 2014).

Patients who suffer an injury while receiving health services, within either the public or the private healthcare sector, can file a claim with the Norwegian System of Patient Injury Compensation (NPE). 3 criteria must be fulfilled for a claim to be accepted:

1. The injury must have been caused during health services (diagnosis, examination, treatment, care, or lack of such), even if no one is to blame. If the injury is severe and unexpected, compensation may be awarded even where no error or omission in treatment has occurred (for example if infection occurs despite adequate prophylaxis).
2. The injury must have caused financial loss to the patient, except if the injury leads to permanent medical impairment of more than 15%, in which case compensation might be awarded despite financial loss. This might be relevant for retired patients or for patients who can continue to work in spite of the disability.
3. The patient must file a claim within a reasonable time (currently set at 3 years) after the patient realizes that the injury is caused by the treatment or lack of treatment received. The claim is filed with NPE at no cost to the patient.

There is compelling evidence that low surgical volume increases the risk of complications and revision after knee arthroplasty surgery (Jaeschke et al. 1989, Badawy et al. 2013, Pamilo et al. 2015, Badawy et al. 2017). Whether this association is also true for injury compensation has not been studied. We evaluated the claims following primary and revision knee arthroplasty surgery filed with NPE and compared the findings with the results from NAR with a focus on annual hospital procedure volume.

## Patients and methods

Data from NAR was collected for the study period (2008 through 2018). The data was stratified by the number of arthroplasty procedures performed annually per hospital. The hospitals were then divided into quarters according to average annual procedure volume. The lowest quarter (Q1) represented 6 institutions with ≤ 57 knee arthroplasty procedures per year. The 2nd quarter (Q2) consisted of 8 institutions with an annual volume of 58–168 procedures, the 3rd quarter (Q3) included 8 institutions with an annual volume of 169–304 procedures, and finally the 4th quarter (Q4) contained 7 institutions with 305 or more knee arthroplasty procedures per year.

All claims filed with NPE following knee arthroplasty surgeries that were performed during the study period were collected. The data were stratified by institution, the patient’s age, sex, type of complication, and any reoperations. The reason for the claim was recorded, together with the decision made by NPE (accepted or rejected claim).

### Statistics

Continuous variables are presented as mean, median, 95% confidence interval (CI), range, and standard deviation (SD), while categorical data is presented in frequencies. Groups were hence compared using the 2-sample independent t-test or the chi-square test. We compared the institutions by procedure volume using ANOVA after asserting conditions were met, and p-values adjusted for multiple testing by Tukey’s comparison test. Associations were quantified by odds ratio. A p-value < 0.05 was considered statistically significant. The analysis was performed using the Statistical Package for Social Sciences (SPSS) version 25 (IBM Corp, Armonk, NY, USA).

### Ethics, funding, and potential conflicts of interest

The Regional Ethical Committee (REK) has deemed approval not necessary as all data are based on already anonymized records (REK 15.10.10). This study received no external funding. The authors declare no conflicts of interests.

## Results

During the study period 2008–2018, 64,241 knee arthroplasty procedures were reported to NAR. There were 59,109 primary knee arthroplasties, of which 6,788 (12%) were unicompartmental knee arthroplasties (UKA). There were 5,132 (8%) revision arthroplasties.

NPE received 572 claims for treatment injuries related to arthroplasties performed during these years, representing 0.9% of all knee arthroplasty procedures. The average age at the time of surgery for the claimants was 62 (range 24–86) years, and 57% of the claims were filed by women (Table 1).

**Table 1.** Demography of knee arthroplasty procedures reported to the Norwegian Arthroplasty Registry (NAR) and compensation claims filed with the Norwegian System of Patient Injury Compensation (NPE) during 2008–2018

Factor	Knee procedures reported to NAR n = 64,241	Compensation claims filed to NPE n = 572	Accepted claims n = 312 (54.5%)	Rejected claims n = 260 (45.5%)	p-value **^a^**
Mean age (SD)	68 (9.7)	62 (10)	63 (9.9)	61 (11)	
range	12–101	24–86	38–86	24–85	
Females, n (%)	38,352 (60)	324 (57)	169 (54)	155 (60)	< 0.001

SD, standard deviation.

**^a^** Chi-square test comparing proportion of women reported to NAR with proportion of women filing a complaint to NPE.

312 (55%) claims were accepted, representing 0.5% of all knee arthroplasties reported to NAR in the period. 259 of the claims were accepted following a primary TKA (0.5% of all primary TKAs) and 25 claims were accepted following a UKA (0.4% of all UKAs, p = 0.2). 11 claims were accepted after revision arthroplasties (0.2% of all revisions) (Table 2).

**Table 2.** Distribution of implant type among 312 accepted compensation claims from the Norwegian System of Patient Injury Compensation during 2008–2018

Implant type	Number (%)
Primary total knee arthroplasty	
cemented	225 (72)
non-cemented	23 (7)
hybrid technique	21 (7)
Primary unicondylar knee arthroplasty	
cemented	21 (7)
non-cemented	4 (1)
Primary patellofemoral arthroplasty	
cemented	1 (0.3)
non-cemented	1 (0.3)
Secondary total knee arthroplasty	
cemented	9 (3)
hybrid technique	2 (0.6)
Secondary patellofemoral arthroplasty	
cemented	1 (0.3)
Other, unspecified	4 (1)

The most common reason for accepted claim was a hospital-acquired infection, in 87 (28%) patients, followed by misplaced implant (81 patients, 26%), and aseptic loosening (40 patients, 13%). Nearly 9% of the claims were accepted due to the wrong indication (Table 3).

**Table 3.** Reasons for accepted claims for treatment injuries following knee arthroplasty surgeries in Norway during 2008–2018. Values are count (%)

	All cases	TKA	UKA	PFA
Reason for accepted claim	n = 312	n = 284	n = 25	n = 3
Hospital-acquired infection	87 (28)	75 (26)	11	1
Misplaced implant	81 (26)	77 (27)	4	–
Early aseptic loosening **^a^**	40 (13)	37 (13)	3	–
Wrong indication	27 (9)	23 (8)	3	1
Wrong choice of implant	12 (4)	10 (4)	2	–
Peroperative nerve injury	12 (4)	11 (4)	–	1
Inadequate follow-up	10 (3)	10 (4)	–	–
Peroperative vascular injury	10 (3)	10 (4)	–	–
Peroperative tendon/				
ligament injury	9 (3)	8 (3)	1	–
Wrong or inadequate				
antithrombotic prophylaxis	4 (1)	4 (1)	–	–
Peroperative fracture	4 (1)	4 (1)	–	–
Nerve injury due from				
bandage/cuff	3 (1)	3 (1)	–	–
Pain	3 (1.0)	3 (1)	–	–
Delayed treatment	3 (1.0)	3 (1)	–	–
Wrong technique	3 (1.0)	2 (0.5)	1	–
Radial nerve damage from				
patient positioning	1 (0.3)	1 (0.3)	–	–
Burn injury from diathermic plate	1 (0.3)	1 (0.3)	–	–
Decubitus from inadequate				
postoperative care	1 (0.3)	1 (0.3)	–	–
Other	1 (0.3)	1 (0.3)	–	–

TKA, total knee arthroplasty; UKA, unicompartmental knee arthroplasty; PFA, patellofemoral arthroplasty.

**^a^** Within 3 years.

15 of 27 claims from private institutions were accepted, compared with 248 (46%) of 545 claims from public hospitals (p = 0.9).

2 claims involving fatalities were recorded, both related to thrombosis prophylaxis. A 78-year-old male undergoing a primary cemented TKA was given low molecular weight heparin while also taking celecoxib, and died from a bleeding gastric ulcer. A 64-year-old male with known increased thrombotic risk received a hybrid primary TKA. He did not receive guideline anticoagulation, and died of acute cerebral stroke. In both cases NPE granted compensation to the survivors.

### Hospital procedure volume

The lowest volume hospitals (< 57 arthroplasties per year, Q1) had a statistically significantly larger fraction of accepted claims per procedures compared with other institutions (Figure). The odds ratio for receiving compensation after a knee arthroplasty performed in a low-volume hospital (Q1) was 3 (CI 2–5) compared with surgery performed in a hospital with higher procedure volume (Table 4). There were no statistically significant differences in accepted claims per annual procedure volume between the other 3 procedure volume quarters (Q2–Q4).

**Figure:**
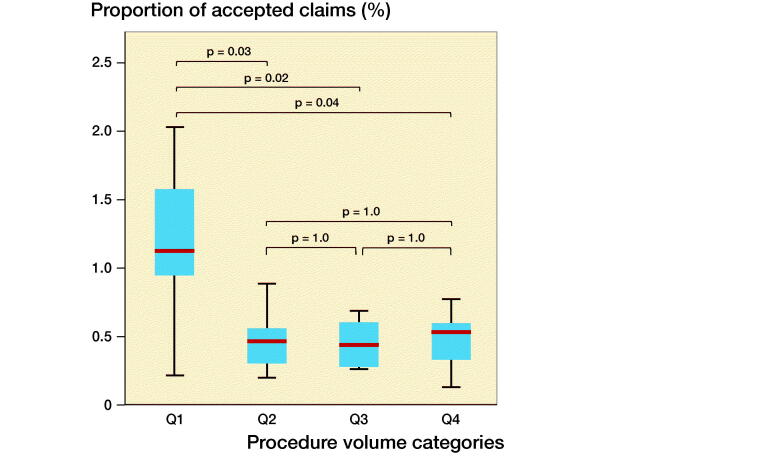
Proportion of accepted claims by number of surgeries stratified by annual hospital procedure volume. The 4 categories represent quarters, see Table 4. P-values derived from ANOVA adjusted with Tukey’s comparison test.

**Table 4.** Risk of accepted compensation claims from the Norwegian System of Patient Injury Compensation during 2008–2018 by annual procedure volume divided into quarters

Quarter (Q)	Odds ratio (95% CI)
Q1 vs, all others	3.0 (2.0–4.5)
Q1 vs. Q2	2.7 (1.7–4.4)
Q1 vs. Q3	2.6 (1.7–4.1)
Q1 vs. Q4	3.4 (2.2–5.2)
Q2 vs. Q3	1.0 (0.7–1.4)
Q2 vs. Q4	1.3 (0.9–1.8)
Q3 vs. Q4	1.3 (1.0–1.7)

Q1, ≤ 57 annual procedures;

Q2, 58–168 annual procedures;

Q3, 169–304 annual procedures; Q4, ≥ 305 annual procedures.

CI, confidence interval.

When considering all claims filed, the lowest volume quarter (Q1) had a similar ratio of accepted claims compared with the 2 highest volume quarter (Q3 and Q4). The 2nd quarter (Q2) had a lower ratio of accepted claims compared with Q1 and Q3 (Table 5).

**Table 5.** Proportion of accepted claims (accepted claims/total claims) for each quarter of annual procedure volume

Quarter	Accepted/ total claims (%)
Q1	26/41 (63)
Q2	47/112 (42) ^a^
Q3	86/159 (54)
Q4	153/260 (59)
Total	312/572 (55)

Q1–Q4, see Table 4

**^a^**Omnibus chi-square statistic was significant at p = 0.02. Analysis of adjusted standardized residuals revealed Q2 to be the main contributor with a z-score of 3.0.

## Discussion

Our main finding is that patients operated on in the lowest volume hospitals have a 3-fold risk of being granted a compensation claim following knee arthroplasty surgery compared with patients being treated in higher volume institutions. Overall, about 0.5% of knee arthroplasty patients were compensated by NPE due to treatment failure.

The main reason for compensation was hospital-acquired infection. This is also the most common reason for accepted claims after other elective knee procedures, such as anterior cruciate ligament reconstruction and cartilage surgery (Randsborg et al. 2018, Aae et al. 2020). The proportion of accepted claims due to infection was particularly high for UKA compared with TKA (p = 0.06). Nearly 60% of knee arthroplasties reported to NAR are performed on women, yet only 57% of compensation claims were filed by women. This small but statistically significant difference might be explained by the fact that men have a 2-fold increased risk of revision due to deep infection (Badawy et al. 2017). Normally, an error in the healthcare provided is needed for a claim to be accepted. However, an exception is made for severe and rare complications, even if no treatment error has been identified. This explains the high rate of compensation following infection and early (within 3 years) aseptic loosening found in our study.

From a surgical point of view, it is interesting that misplaced implant is the 2nd greatest reason for compensation, representing over a quarter of the accepted claims. More claims from patients treated at the lowest volume hospitals may relate to the fact that both surgical and hospital volume affects clinical results and complication rates (Soohoo et al. 2006, Paterson et al. 2010, Badawy et al. 2013, Pamilo et al. 2015). Furthermore, it is worth noting that nearly 9% of the claims were accepted due to the wrong indication. The relative success of knee arthroplasty must not lead clinicians to utilize a one-size-fits-all solution for knee pain. The proportion of claims accepted due to the wrong indication or wrong choice of implant was particularly high for UKA compared with TKA, but did not reach statistical significance. Nevertheless, our findings serve as a reminder that nonoperative measures and correlation between radiological findings and clinical symptoms remain cornerstones in the indication for knee arthroplasty (Schmitt et al. 2017). Unfortunately, avoidable treatment injuries such as wrong indication or inadequate follow-up remain major reasons for accepted claims in orthopedic surgery (Randsborg et al. 2018, Aae et al. 2020). These are modifiable factors and demonstrate how compensation claim reports can be useful for clinicians to learn from other people’s mistakes. By focusing on proper indication, surgical technique, and follow-up routine, the number of adverse outcomes and thus compensation claims will likely be reduced.

Another interesting finding in our study is that the risk of compensation following primary UKAs was not higher than for primary TKAs. This came as some surprise to us, because the revision rate for UKAs is higher than for TKAs (Chawla et al. 2017, Arias-de la Torre et al. 2019, Jennings et al. 2019). However, the likelihood of compensation is not identical to the risk of complication. For a claim to be accepted, a treatment failure must have occurred. A revision of a UKA does not necessary indicate that the primary surgery was a treatment error. There is a lower threshold for surgeons to revise a painful UKA (Johnson et al. 2020), which may explain the higher rate of revision of UKA, but no difference in likelihood of treatment injury compensation.

Procedure volume has been a hot topic since 1979, when Luft et al. asked the simple question: Should operations be regionalized? The effect on both hospital and surgeon volume on adverse outcome has been discussed across medical fields since then, and there is little doubt that very low volume increases risk of adverse outcome. For knee arthroplasties in particular, several authors have concluded that surgery performed in a low-volume hospital increases the risk of adverse outcome (Soohoo et al. 2006, Paterson et al. 2010, Badawy et al. 2013, Pamilo et al. 2015). Our study confirms that the likelihood of compensation due to treatment injury following knee arthroplasty is also increased in low-volume institutions. In support of this, a report from Finland found that hospital volumes of less than 200 annual arthroplasty procedures were associated with more compensated treatment errors (Jarvelin et al. 2012). However, 200 procedures per year would in our study place the hospital in the 2nd highest quarter. Therefore, our study provides a more detailed analysis of medium institutional procedure volume. Notably, only the lowest quarter (< 57 procedures per year) had a significant increased likelihood of accepted claims.

### Limitations

There are several limitations to this study. Some patients, who underwent surgery towards the end of the study period, may not yet have filed a compensation claim. There could be regional and institutional differences in the culture of claiming compensation or the information given to patients concerning the possibility of filing a complaint. It is also likely that some treatment errors were never reported to NPE. Furthermore, individual surgeons’ annual procedure volume was not available, which could influence results. There could be some variation in annual procedure volume during the study period causing fluctuation between quarters.

Our data is collected from a single country, with a public compensation scheme based on the principle of no blame. This is similar to systems in other Nordic countries, but different from countries such as the United Kingdom, the United States, Italy, and Germany that have a tort-based system. This may limit the generalizability of our study. However, our purpose was not to compare different compensation schemes, but to analyze the causes and aspects of compensation following knee arthroplasty surgery. It is important to point out that this is not a study on complications following knee arthroplasty. Most complications will never be reported to NPE. A review of compensation claims investigates the quality of the healthcare provided, not the outcome of the medical procedures in question.

In summary, the overall likelihood of ending up with compensation due to treatment error following knee arthroplasty was 0.5%. The likelihood was 3 times greater for patients operated on in the lowest volume hospitals.
